# A Review of *ex vivo* Elemental Mapping Methods to Directly Image Changes in the Homeostasis of Diffusible Ions (Na^+^, K^+^, Mg^2 +^, Ca^2 +^, Cl^–^) Within Brain Tissue

**DOI:** 10.3389/fnins.2019.01415

**Published:** 2020-01-22

**Authors:** David Hartnell, Wendy Andrews, Nicole Smith, Haibo Jiang, Erin McAllum, Ramesh Rajan, Frederick Colbourne, Melinda Fitzgerald, Virginie Lam, Ryusuke Takechi, M. Jake Pushie, Michael E. Kelly, Mark J. Hackett

**Affiliations:** ^1^School of Molecular and Life Sciences, Faculty of Science and Engineering, Curtin University, Perth, WA, Australia; ^2^Curtin Health Innovation Research Institute, Curtin University, Perth, WA, Australia; ^3^Curtin Institute for Functional Molecules and Interfaces, Curtin University, Perth, WA, Australia; ^4^School of Molecular Sciences, Faculty of Science, University of Western Australia, Perth, WA, Australia; ^5^Melbourne Dementia Research Centre, Florey Institute of Neuroscience and Mental Health, Parkville, VIC, Australia; ^6^Biomedicine Discovery Institute, Monash University, Clayton, VIC, Australia; ^7^Department of Neuroscience and Mental Health Institute, University of Alberta, Edmonton, AL, Canada; ^8^Department of Psychology, Faculty of Arts, University of Alberta, Edmonton, AL, Canada; ^9^School of Biological Sciences, University of Western Australia, Perth, WA, Australia; ^10^Perron Institute for Neurological and Translational Science, Perth, WA, Australia; ^11^School of Public Health, Faculty of Health Sciences, Curtin University, Perth, WA, Australia; ^12^Department of Surgery, College of Medicine, University of Saskatchewan, Saskatoon, SK, Canada

**Keywords:** ischemia, metabolism, imaging, XFM, SIMS, LA-ICP-MS, PIXE, microprobe

## Abstract

Diffusible ions (Na^+^, K^+^, Mg^2+^, Ca^2+^, Cl^–^) are vital for healthy function of all cells, especially brain cells. Unfortunately, the diffusible nature of these ions renders them difficult to study with traditional microscopy *in situ* within *ex vivo* brain tissue sections. This mini-review examines the recent progress in the field, using direct elemental mapping techniques to study ion homeostasis during normal brain physiology and pathophysiology, through measurement of ion distribution and concentration in *ex vivo* brain tissue sections. The mini-review examines the advantages and limitations of specific techniques: proton induced X-ray emission (PIXE), X-ray fluorescence microscopy (XFM), secondary ion mass spectrometry (SIMS), laser-ablation inductively coupled plasma mass spectrometry (LA-ICP-MS), and the sample preparation requirements to study diffusible ions with these methods.

## Introduction

The cations of alkali (Na^+^, K^+^) and alkaline (Mg^2+^, Ca^2+^) earth metals, in addition to anions such as Cl^–^, are essential to cell physiology. Cell membrane potential for example, is maintained by ion pumps that carefully control intra-cellular and extra-cellular concentrations of Na^+^, K^+^, Mg^2+^, Ca^2+^, and Cl^–^ (Hansen and Zeuthen, 1981; Hansen, 1985). In addition, cell volume is regulated by osmotic pressure, for which tightly controlled intra-cellular and extra-cellular ion concentrations are essential (Hansen and Zeuthen, 1981; Hansen, 1985; Stys, 1998; Malek et al., 2003). In the brain, glial cells constantly monitor and adjust extra-cellular ion concentrations in order to maintain cell membrane potential and osmotic pressure (Simard and Nedergaard, 2004; Abbott et al., 2006). Glia can regulate extra-cellular ion concentration through their control of influx or efflux of ions across the blood-brain barrier or ventricle-brain barrier (Walz, 1989; Simard and Nedergaard, 2004; Abbott et al., 2006; Mack and Wolburg, 2013). Dysfunction of barrier capacity can therefore, lead to ion dysregulation (Walz, 1989; Simard and Nedergaard, 2004; Abbott et al., 2006; Mack and Wolburg, 2013). Detection of altered ion homeostasis is thus, an important marker of cell health and cell physiology, can be diagnostic of pathological conditions (e.g., ischemia, cell depolarization), and could be used to monitor therapeutic efficacy of strategies designed to restore homeostasis.

Diffusible ions also perform specialized functions in brain nerve cells, e.g., neurotransmission. The diffusible nature of the aforementioned ions (Na^+^, K^+^, Mg^2+^, Ca^2+^, Cl^–^) enables communication along nerve cells in the form of electrical currents (action potentials). The brain has evolved to use a number of neurotransmitter systems (e.g., acetylcholine, glutamate, GABA), upon which neurotransmitter-to-receptor binding opens ion channels in cell membranes. The opening of ion channels results in rapid diffusion of ions across the intra-cellular/extra-cellular concentration gradient, thus generating an action potential. Elegant approaches have been developed to study electrical impulses generated by ion influxes during neuron and glial signaling (e.g., electrophysiology), or to directly image changes in specific ionic gradients *in vitro* (e.g., intra-cellular Ca^2+^ sensitive fluorescence sensors in living organotypical tissue slice models, or *in vivo* through cranial windows). Direct measurement of ionic homeostasis within tissues, in animal models of brain injury or pathology has been more difficult to achieve.

**FIGURE 1:**
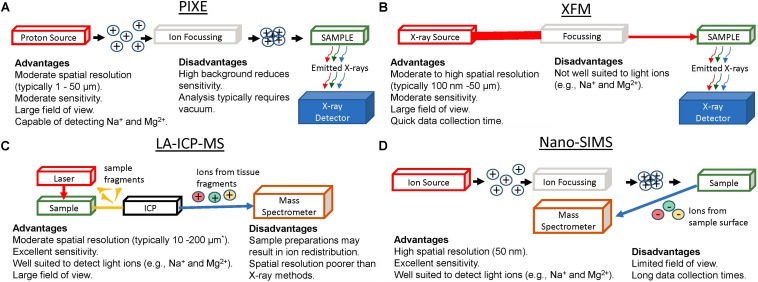
Graphical Representation of apparatus for elemental mapping using: PIXE **(A)**, XFM **(B)**, LA-ICP-MS **(C)**, and Nano-SIMS **(D)**, highlighting major advantages and limitations of each method. ^∗^Recent developments in LA-ICP-MS now enable capability for lateral resolutions ranging from 200 nm to 2 μm.

**FIGURE 2:**
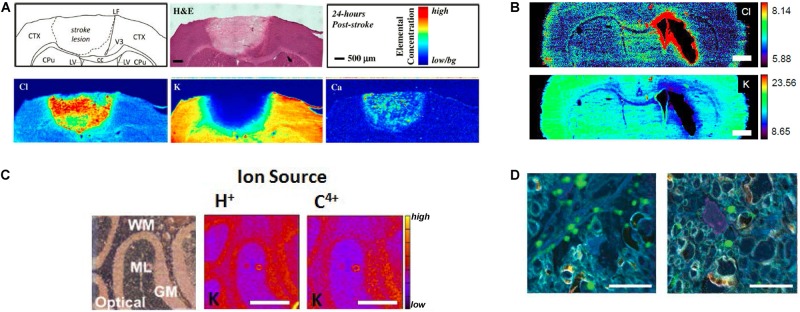
**(A)** XFM elemental mapping of ion homeostasis after ischemic stroke. Clockwise from top left: Schematic of brain regions affected by stroke; H&E histology of brain tissue 24 h after ischemic stroke; relative concentration and scale bar; Ca influx is observed 24 h after ischemic stroke; K efflux is after ischemic stroke; Cl influx is observed after ischemic stroke. **(B)** XFM elemental mapping of Cl and K distribution after hemorrhagic stroke: top panel shows Cl influx around swollen lateral ventricles; bottom panel shows K efflux. Scale bar = 1 mm. **(C)** PIXE elemental mapping of K distribution in 10 μm thick sections of mouse cerebellum, showing optical bright field image of unstained tissue (left), PIXE elemental map for K determined with a H^+^ source (center), and PIXE elemental map for K determined with a C^4+^ source (right). Scale bar = 500 μm. **(D)** Nano-SIMS imaging of Ca microdomains in optic nerve tissue. Left panel shows Ca microdomains (green) in control optic nerve, right panel shows Ca microdomains after nerve injury. Scale bar = 10 μm. Figures adapted with permission from references X **(A)**, Y **(B)**, Z **(C)**. **(A)** Reprinted with permission from Chwiej et al. (2011) Copyright 2018 American Chemical Society. **(B)** Reproduced with permission from Williamson et al. (2016). **(C)** Reprinted from Lee et al. (2013) with permission from Elsevier. **(D)** Reproduced from Niesporek et al. (2017) permission of The Royal Society of Chemistry.

Much of our knowledge on the role of ions in brain function has come from: observational studies on the effect of ion deprivation or ion overload in cell or tissue culture models; genetic modification of ion channels and observation of the effects on cell function; or bulk elemental analysis of cells, or fluids (e.g., ion selective electrodes or micro-dialysis of extra-cellular fluid) following perturbation of brain physiology, often achieved with agonist or antagonists that activate or deactivate ion channels or receptors. Ion homeostasis can be directly monitored *in vivo* using NMR e.g., ^35^Cl and ^23^Na (Veniero and Gupta, 1992; Bachelard and Badar-Goffer, 1993; Niesporek et al., 2017), but spatial resolution is limited with typical voxel sizes on the order of hundreds of microns. Only recently, however, have sophisticated elemental mapping techniques become available to directly study ion distributions in tissues. This work is now yielding important new insight into the pathophysiology of brain damage and neurodegenerative disease. Direct elemental mapping techniques include X-ray Fluorescence Microscopy (XFM), Proton Induced X-ray Emission (PIXE), Laser Ablation Inductively Coupled Plasma Mass Spectrometry (LA-ICP-MS), and Secondary Ion Mass Spectrometry (SIMS). These techniques (shown schematically in Figure 1), have enabled neuroscientists to directly study the distribution of Na^+^, K^+^, Mg^2+^, Ca^2+^, and Cl^–^, *in situ* within *ex vivo* tissue sections at cellular and sub-cellular spatial resolution, in conditions such as traumatic brain injury (Chwiej et al., 2011; Lozić et al., 2014), ischemic stroke (Caine et al., 2016; Pushie et al., 2018), hemorrhagic stroke (Hackett et al., 2015b; Williamson et al., 2016), schizophrenia (Lins et al., 2016), and epilepsy (Inamura et al., 1990; Ren et al., 1999; Chwiej et al., 2008; Chwiej et al., 2012a), to name a few (Figure 2). This mini-review highlights the recent applications of XFM, PIXE, LA-ICP-MS, and SIMS, to study the role of ions in healthy brain function and during disease or injury. Specific considerations for sample preparation will be discussed, and although detailed theory behind the analytical techniques and instrumentations are beyond the scope of this mini-review, we hope the citations contained herein will serve as a valuable guide.

## Discussion

### Sample Preparation

Sample preparation is an important consideration for *ex vivo* imaging, including all the analytical methods described in this article. Practically, it is impossible for *ex vivo* analysis to completely reproduce the *in vivo* condition. The overarching aim of sample preparation for *ex vivo* analyses should therefore, be to best preserve the *in vivo* chemical composition and distribution. In some cases, where substantive changes in chemical composition or distribution may occur, analyses may still be valid if the results from *ex vivo* analysis are at least proportional to the *in vivo* condition. These considerations are especially important for *ex vivo* analyses of concentration and distribution of diffusible ions, which are easily re-distributed or leached from the sample by aqueous reagents used during perfusion or chemical fixation (Chwiej et al., 2005; Hackett et al., 2011a). Another consideration, when using animal models, is the post-mortem interval. The time and temperature between animal euthanasia or spontaneous death and tissue harvest may be a critical consideration in light of the ischemic conditions and metabolic alterations that occur in brain tissue following cessation of blood flow (Shank and Aprison, 1971; Petroff et al., 1988; Hackett et al., 2015a). Likewise, the choice of anesthetic can have an impact on neuron activity, affecting brain metabolism and potentially ionic gradients (Makaryus et al., 2011). Further, the response to anesthesia, in addition to autolytic processes that occur after cell death should not be assumed to be consistent between healthy or control experimental groups and various disease states.

Most studies in this field advocate for the use of flash-frozen brain tissue when studying diffusible ions, avoiding the use of perfusion or chemical fixatives (Chwiej et al., 2005; Matsuyama et al., 2010; Hackett et al., 2011a; Perrin et al., 2015; Jin et al., 2017). The time-period over which the tissue freezes is an important consideration as damage due to ice-crystal formation may be significant, thus altering the level of cellular or sub-cellular detail that can be preserved in the sample. Generally, rapid flash freezing in liquid nitrogen-cooled isopentane will achieve faster freezing and therefore better tissue preservation compared with liquid nitrogen alone, or freezing on dry ice. For the most immediate cessation of brain biochemistry following animal death, *in situ* brain freezing or decapitation into liquid nitrogen is essential, as ischemic and autolytic processes occur to a greater extent in the time period during brain removal and tissue dissection (Lust et al., 1973; Veech et al., 1973; Hackett et al., 2015a). If the former approach is used, the frozen brain tissue must be extracted from the frozen animal skull, which is typically achieved by chiseling out the brain at dry ice temperatures (Lust et al., 1973; Veech et al., 1973; Hackett et al., 2015a). Such a procedure, although offering more optimal preservation of brain biochemistry is considerably more time consuming, and the choice between the methods should be made with consideration of the specific experimental aims and expected outcomes.

Prior to imaging analyses, cryo-sections are typically cut at a temperature of −15 to −25°C, and typical section thickness is 1–50 μm. To achieve sub-cellular resolution imaging section thickness of <5 μm is often necessary (Hackett et al., 2016), with cellular detail well preserved in sections 5–20 μm thick (Chwiej et al., 2008, 2011, 2012a,b; Hackett et al., 2015b; Caine et al., 2016; Lins et al., 2016; Williamson et al., 2016). Sections 50 μm or thicker are more suitable for regional or sub-regional analyses (Popescu et al., 2009; Pushie et al., 2011). Tissue sections are often analyzed dehydrated (air-dried or freeze-dried) (Chwiej et al., 2008, 2011, 2012a,b; Hackett et al., 2015b; Caine et al., 2016; Lins et al., 2016; Williamson et al., 2016), however it has been shown that analyses of frozen samples that do not thaw at any point during sample preparation is required to best preserve sub-cellular elemental distribution on the sub-micron scale (Matsuyama et al., 2010; Roudeau et al., 2014).

### Applications of PIXE to Study Brain Ion Homeostasis

Excellent descriptions of the theory and application of Proton (or particle) induced X-ray emission (PIXE) to elemental mapping are contained within numerous reviews. Although detailed explanation of the technique is beyond the scope this mini-review, in brief: PIXE arises from ionization of an atom through ejection of a core electron (e.g., 1s) to yield a core-hole. The core-hole is subsequently filled via relaxation of an outer shell electron, which releases an X-ray with an energy characteristic of the relaxation process involved. The energy of the emitted X-ray photon, therefore, is proportional to the stabilization energy gained when an outer shell electron fills the inner core-hole. This process fundamentally differs from X-ray fluorescence microscopy (XFM, discussed in next section) as the core hole is generated by an ion beam as opposed to an incident photon (X-ray) absorption. This underlying difference gives rise to an inherent relative increase in sensitivity of PIXE measurements relative to XFM, for detection of lighter elements, such as diffusible ions (Na^+^, K^+^, Mg^2+^, Ca^2+^, and Cl^–^) (Lindh, 1990). Unfortunately, the rapidly decelerating ion beam also produces a substantial background signal, also known as Bremsstrahlung radiation, which can complicate quantitative analyses (Ryan, 2000; Ryan et al., 2002; Lee et al., 2013). The spatial resolution obtainable with PIXE is largely limited by repulsive forces associated with focusing an ion beam, and therefore is not capable of the same level of spatial resolution as XFM. Spatial resolution for PIXE is typically on the order of microns (1–50 μm), as reviewed by others (Paunesku et al., 2006; Petibois and Guidi, 2008; Qin et al., 2011; Pushie et al., 2014). Therefore, while PIXE does not offer the same spatial resolution or detection limits as can be achieved with XFM for routine experiments (Petibois and Guidi, 2008; Qin et al., 2011), PIXE is well suited to detect ions at the concentrations found during normal and abnormal physiological conditions within the brain (Kemp and Danscher, 1979; Ren et al., 1999; Hackett et al., 2011b; Lee et al., 2013). PIXE has made important contributions to our understanding of ion dyshomeostasis within hippocampal neurons suffering excitotoxicity. Specifically, PIXE was used to reveal Ca^2+^ accumulation and K^+^ efflux within hippocampal neuronal layers following membrane depolarization in conditions such as brain ischemia (Martins et al., 1988; Tsuda et al., 1989), and epilepsy (Inamura et al., 1990; Ren et al., 1999). Indeed, direct elemental mapping measurements made using PIXE were critical to validate the excitotoxicity and mitochondrial Ca^2+^ overload hypothesis that were proposed to drive selective neurodegeneration that occurs 1–3 days following brain ischemia and epilepsy (Martins et al., 1988; Siesjö and Bengtsson, 1989). Interestingly, although PIXE offers capability to detect lighter ions such as Na^+^ and Mg^2+^, this has been rarely reported for brain tissue however, application to other biological questions/systems e.g., Na^+^ and Mg^2+^ in erythrocytes of children suffering cystic fibrosis, has occurred (Lindh, 1990).

### Applications of XFM to Study Brain Ion Homeostasis

In contrast to PIXE, XFM is dependent on the creation of a core hole through absorption of an X-ray photon, which promotes a core electron to an unoccupied orbital (resonant absorption) or the continuum (ionization). Detailed reviews on the theory and instrumentation of XFM for elemental mapping are found within the references below. The diffraction-limited spatial resolution that can be achieved with the short wavelength of X-rays used for XFM is on the nano scale, but this is rarely achieved (see following review articles for further details) (Paunesku et al., 2006; Petibois and Guidi, 2008; Qin et al., 2011; Pushie et al., 2014). Typical optical components for XFM experiments provide spatial resolution on the order of 10–100 μm (using a capillary or pin hole), ∼1–2 μm (with Kirkpatrick-Baez mirrors), or ∼100–200 nm (Frenzyl zone plates) (Paunesku et al., 2006; Petibois and Guidi, 2008; Qin et al., 2011; Pushie et al., 2014). This range of XFM optical configurations is well suited to monitor elemental distribution at the tissue or tissue sub-region level (capillary or pin hole), cellular level (Kirkpatrick-Baez mirrors), and sub-cellular level (Frenzyl zone plate) (Paunesku et al., 2006; Petibois and Guidi, 2008; Qin et al., 2011; Pushie et al., 2014).

Emitted X-ray fluorescence from diffusible ions (Na^+^, K^+^, Mg^2+^, Ca^2+^, and Cl^–^) occurs over the energy range 1000–3000 eV and photons in this relatively low-energy range impose technical limitations for both PIXE and XFM measurements. The Be window on energy dispersive X-ray detectors, such as Si drift detectors absorb much of the emitted X-ray fluorescence across this energy range. Further, Ar_(g)_, which composes ∼0.93% of air, as well as the variable amount of water vapor (humidity) and sample-to-detector pathlength will all attenuate much of the emitted X-ray fluorescence signal at these low energies (Pushie et al., 2014). Due to these challenges the use of vacuum or He_(g)_-filled sample environments for PIXE and XFM experiments may be required (Lins et al., 2016). Although this adds to the complexity of the experimental setup, added considerations for accommodating instrumentation, vacuum or He sample environments can be accommodated on most PIXE or XFM experimental end-stations. In practice, analysis of Cl^–^, K^+^, Ca^2+^ is routinely achieved on XFM and PIXE instrumentation (Chwiej et al., 2008, 2011, 2012a,b; Hackett et al., 2011b, 2015b; Caine et al., 2016; Lins et al., 2016; Pushie et al., 2018). Imaging Na^+^ and Mg^2+^ present significant challenges that cannot be overcome for XFM measurements on biological specimens, including poor penetration/escape depth for the X-ray fluorescence signal arising from the sample as well as relatively poor fluorescence yields compared with heavier elements.

Both XFM and PIXE techniques offer simultaneous elemental detection, which has provided the capability to quantify multiple ion types in brain tissue. Recently, XFM has been extensively applied to characterize alterations in ion homeostasis (K^+^, Ca^2+^, Cl^–^) that occur in experimental rodent models of ischemic stroke (Caine et al., 2016; Pushie et al., 2018) and hemorrhagic stroke (Hackett et al., 2015b; Williamson et al., 2016). Specifically, it has recently been shown that the loss of K^+^ from neurological tissue and the large influx of Cl^–^ and Ca^2+^ can be used to differentiate between the ischemic infarct (containing dead or dying brain tissue that has undergone irreparable metabolic changes), the ischemic penumbra (tissue that can potentially be rescued), and healthy tissue, following stroke (Figure 2A; Caine et al., 2016; Pushie et al., 2018). One of the ongoing challenges in the stroke community has been identification of the size and location of “penumbra” tissue after ischemic stroke (Figure 2A) or peri-hematoma zone after hemorrhagic strok (Figure 2B). Penumbra (or peri-hematoma) tissue contains neurons that although partially affected by the stroke, are recoverable (i.e., not dead tissue or cells destined to die). The stroke field continues to seek agents or approaches that will reduce cell death in the penumbra (or peri-hematoma zone) after stroke to minimize the delayed loss of brain function that occurs in stroke survivors. Thus, the capability to accurately identify the stroke penumbra based on ionic homeostasis is expected to find growing use in this research field.

Epilepsy shares several similarities with stroke pathology; specifically, over-excitation of neurons (excitotoxicity) is a key driver of cell damage during seizures or after ischemic stroke. Ca^2+^ overload and intracellular Ca^2+^ accumulation is an important marker of excitotoxicity. Chwiej and colleagues have applied XFM to characterize Ca^2+^ accumulation in hippocampal sub regions (e.g., CA3 mossy fibers) following epileptic seizures in rodent models (Chwiej et al., 2008; Chwiej et al., 2012a, b), and after therapeutic intervention (Chwiej et al., 2010).

In contrast to stroke and epilepsy, very little neurodegeneration, if any, is observed in schizophrenia. Rather, the condition appears to arise from altered neuron connections, changes to white matter and disturbed cellular signaling. Altered ionic homeostasis is observed clinically in human schizophrenia, which includes calcifications within brain ventricles (Bersani et al., 1999; Marinescu et al., 2013). Recently, XFM has been used to identify micro-calcifications in a rodent model of maternal inflammation, a key risk factor for schizophrenia (Lins et al., 2016). In addition to micro-calcifications, the study observed elevated Cl^–^ levels in brain parenchyma surrounding the lateral ventricles (Lins et al., 2016). The finding provides evidence of a direct link through which neurotransmitter imbalances (namely serotonin) may alter neuronal communication along an axis of disturbed ion homeostasis, which is now being studied in greater detail.

### Applications of LA-ICP-MS to Study Brain Ion Homeostasis

Mass spectrometry offers an inherent advantage to detect lighter ions such as Na^+^ and Mg^2+^, relative to PIXE and XFM, as the spectrometric detection concerns mass to charge ratios, not photon energy. Therefore, the low energy of X-ray emission from lighter elements that is a limitation for PIXE and XFM measurements, is irrelevant for mass spectrometric methods (e.g., LA-ICP-MS and SIMS). The most widely used application of mass spectrometry for elemental mapping is laser ablation inductively coupled plasma mass spectrometry (LA-ICP-MS) (Jurowski et al., 2013; Becker et al., 2014; Paul et al., 2015). The spatial resolution of LA-ICP-MS is defined by the ablative area of the focused laser (typically 10–200 μm) (McRae et al., 2009), which is generally an order of magnitude or more poorer than X-ray techniques (Petibois and Guidi, 2008; McRae et al., 2009; Qin et al., 2011; Becker et al., 2014). Thus, while XFM can be readily used to study diffusible ions at the cellular and sub-cellular level, LA-ICP-MS is generally limited to analyses across different tissue regions or tissue sub-regions, but rarely single cells (Becker et al., 2014; Paul et al., 2015). However, recent developments in the LA-ICP-MS field have enabled lateral resolutions of 200 nm – 2 μm to be obtained, which is expected to open greater opportunities for sub-cellular LA-ICP-MS (McRae et al., 2009; Zoriy and Becker, 2009; Pozebon et al., 2017; Löhr et al., 2018).

Although suitable to the study of diffusible ions, LA-ICP-MS has mainly found widespread use in the study of transition metals in brain tissues (Pozebon et al., 2010; Becker et al., 2014; Paul et al., 2015). This is likely due, in part, to the choice of sample preparation. Typically, brain tissue for LA-ICP-MS measurements has been cryo-protected by first soaking the tissue in a concentrated sucrose solution to avoid tissue damage during subsequent freezing (Paul et al., 2015; Bishop et al., 2018). This is often done to enable multi-modal elemental mapping, histology, and immuno-histochemical characterization of adjacent tissue sections from the same specimen (Paul et al., 2015; Bishop et al., 2018). Unfortunately, such preparations contribute to leaching away or redistributing the majority of diffusible ions from brain tissue, as already discussed (Chwiej et al., 2005; Hackett et al., 2011a). With increased awareness of the sample preparation constraints required to preserve ion concentrations and their distribution in brain tissue, LA-ICP-MS can be expected to gain increasing application in this field. Progress has been made in this respect, with development of laser ablation of frozen-hydrated tissues at cryogenic temperatures, one avenue to negate sample preparation induced elemental redistribution (Feldmann et al., 2002; McRae et al., 2009). Furthermore, the capability of LA-ICP-MS to differentiate between stable isotopes opens possibilities for dose-trace studies, which have immense potential for investigating brain physiology following injury or during disease.

### Applications of Nano-SIMS to Study Brain Ion Homeostasis

Similar to LA-ICP-MS, nanoscale secondary ion mass spectrometry (nano-SIMS) offers the analytical advantages associated with mass spectrometry – excellent detection limits and sensitivity to light elements (e.g., Na^+^, Mg^2+^, K^+^, Ca^2+^, and Cl^–^) (Chandra et al., 2000, 2016; Kim et al., 2008; Jiang et al., 2016). Compared with LA-ICP-MS (and PIXE and XFM), nano-SIMS also offer the opportunity to collect elemental maps at high spatial resolution (down to 50 nm), in combination with high elemental sensitivity (mg kg^–1^), and can detect both the lighter elements as well as most of the heavier elements (Chandra et al., 2000; Chandra et al., 2016; Jiang et al., 2016). Nano-SIMS uses a focused ion beam (predominantly Cs^+^ or O^–^) to scan the sample surface to generate secondary ions for detection, and it routinely achieves elemental mapping to spatial resolution of 50–100 nm (Chandra et al., 2000; Lozić et al., 2014; Chandra et al., 2016). Unfortunately, measurement times are relatively long and only a small field of view can be imaged (50 × 50 μm^2^). In this respect, nano-SIMS complements LA-ICP-MS, with the latter being used for survey scans of larger areas, while the former can be used to probe sub-cellular details. The preservation of distributions of diffusible ions also represents a challenge for nano-SIMS analysis due to its requirement to preserve distributions of diffusible ions at nanoscale. As analysis must be conducted in an ultra-high vacuum, high-pressure freezing is often utilized to rapidly freeze a small volume of tissue, followed by freeze substitution and resin infiltration at low temperatures (e.g., −50°C) to enable nano-SIMS analysis of these diffusible ions (Lozić et al., 2014). The sensitivity of nano-SIMS to lighter elements has leant the technique to investigations of sub-cellular distributions of diffusible ions such as Mg^2+^ and Ca^2+^ in the CNS, in a range of studies that include ischemia (Kim et al., 2008), traumatic injury (Figure 2C; Lozić et al., 2014), and metabolic alterations associated with proliferating brain cancer (Chandra et al., 2000; Chandra et al., 2016). Nano-SIMS detection of diffusible ions together with lanthanide metals conjugated to antibodies can also enable comparisons in immuno-labeling between multiple cellular sub-populations and structures (Giacci et al., 2018), associated with alterations in ionic homeostasis. This latter capability can also be achieved with LA-ICP-MS, and the two techniques together provide the ability to cover the range from sub-cellular (nano-SIMS) to large scale tissue imaging (LA-ICP-MS) (Bishop et al., 2018).

## Conclusion

Although direct elemental mapping techniques have been most readily applied to study transition and heavy metal ions within the CNS, with careful selection of sample preparation protocols they are also well suited to study diffusible ions (Na^+^, Mg^2+^, Cl^–^, K^+^, Ca^2+^), *in situ*, in *ex vivo* tissue sections. Such capability is much needed, due to the important roles such ions play in healthy brain physiology and pathophysiology. The unique advantages and limitations of the various elemental mapping techniques (PIXE, XFM, LA-ICP-MS, nano-SIMS) can be leveraged during experimental design, to develop strategies most suited to target lighter ions (e.g., Na^+^ and Mg^2+^) or heavier ions (Cl^–^, K^+^, Ca^2+^), and for analyses at sub-cellular, or cellular spatial resolution. It is anticipated that increased awareness of these capabilities to study diffusible ions will see further integration of these methods into the “routine-toolbox” available for the modern neuroscientists.

## Author Contributions

All authors contributed to the conceptual design. Specifically, each author is interested in studying diffusible ions in the CNS, during: aging (DH, WA, VL, and RT), ischemic stroke (MP and MK), intra-cerebral hemorrhage (FC), and traumatic brain injury (NS, RR, and MF). HJ, EM, MP, and MH were specifically interested in the development of analytical methodology to study diffusible ions. All authors were involved in the writing and editing of the manuscript, specific author contributions (writing and editing) were: DH: primary Ph.D. student conducting literature review, writing manuscript, and preparing figures. WA: honors student assisted DH in literature review and figure preparation. NS and HJ: expertise input for nano-SIMS. EM: expertise input for PIXE and LA-ICP-MS. RR and MF: expertise input for traumatic brain injury. FC: expertise input for intra-cerebral hemorrhage. RT and VL: expertise input for blood-brain barrier and glial cells. MP and MK: expertise input for ischemic stroke. MH: overall conceptual design for review, supervisor of DH and WA – advising in writing and figure design.

## Conflict of Interest

The authors declare that the research was conducted in the absence of any commercial or financial relationships that could be construed as a potential conflict of interest.
